# Dengue Infection Triggering Concurrent Thrombotic Thrombocytopenic Purpura in a Case of Chronic Idiopathic Thrombocytopenic Purpura

**DOI:** 10.7759/cureus.43684

**Published:** 2023-08-18

**Authors:** Akash Rana, Prashant Ahlawat, Prateek Upadhyay, Akshita Gupta, Aman Bansal

**Affiliations:** 1 General Medicine, Government Medical College & Hospital, Chandigarh, Chandigarh, IND; 2 Anesthesiology, Government Medical College & Hospital, Chandigarh, Chandigarh, IND

**Keywords:** thrombocytopenic purpura, dengue virus infection, itp managment, dengue, idiopathic thrombocytopenic purpura, thrombotic thrombocytopenic purpura

## Abstract

We present a case report detailing the medical history of a 53-year-old female who had a well-established 10-year history of idiopathic thrombocytopenic purpura (ITP). The patient presented with fever and gum bleeding, prompting a series of laboratory investigations. These examinations revealed concurrent thrombocytopenia and hemolytic anemia, alongside a positive test result for serum dengue IgM antibodies. Initial treatment for the patient involved intravenous administration of glucocorticoids and intravenous immunoglobulin. Regrettably, this therapeutic intervention did not yield a favorable response. Subsequent clinical developments, including the onset of generalized tonic-clonic seizures, raised suspicions of thrombotic thrombocytopenic purpura (TTP). A notable diagnostic indicator was the elevated PLASMIC score (platelet count; combined hemolysis variable; absence of active cancer; absence of stem-cell or solid-organ transplant; mean corpuscular volume; international normalized ratio; creatinine), reinforcing the consideration of TTP. To confirm the diagnosis, ADAMTS13 (a disintegrin and metalloproteinase with thrombospondin motifs 13) enzyme levels were assessed and found to be low. Consequently, the patient was diagnosed with TTP. Plasmapheresis was administered, resulting in a positive clinical response after two cycles. Notably, the patient experienced a resolution of thrombocytopenia and hemolytic anemia. Following successful treatment, the patient was discharged with a prescription for immunosuppressants. This case underscores the critical importance of including TTP as a potential differential diagnosis when encountering patients with chronic ITP. TTP is characterized by its acute and life-threatening nature, often deviating from the typical clinical presentation. The application of the PLASMIC score serves as a valuable tool in guiding decision-making processes when TTP is suspected.

## Introduction

Thrombocytopenia represents a frequently encountered condition in clinical practice, with a diverse array of underlying causes, encompassing infections, malignancies, and autoimmune disorders. Among the conditions leading to thrombocytopenia, thrombotic thrombocytopenic purpura (TTP) and idiopathic thrombocytopenic purpura (ITP) emerge as distinct entities. While both conditions share the hallmark of thrombocytopenia, their management strategies and urgency for treatment exhibit notable disparities.

ITP is typified by isolated thrombocytopenia devoid of an identifiable etiology, often linked to platelet-associated IgG or IgM antibodies [1]. A substantial proportion of cases (approximately 80%) are categorized as primary ITP, while secondary ITP stems from an array of underlying factors, including infections, autoimmune disorders, primary immune deficiencies, or malignancies [2]. In contrast, TTP represents a relatively uncommon yet gravely perilous disorder. It arises from an insufficiency of the metalloproteinase ADAMTS13 (a disintegrin and metalloproteinase with thrombospondin motifs 13) enzyme, pivotal for cleaving von Willebrand factor (vWF), an integral component of the coagulation cascade.

Within the context of TTP, the accumulation of large vWF multimers culminates in the formation of platelet-rich thrombi circulating throughout the body, ultimately yielding thrombocytopenia, microangiopathic hemolytic anemia, and compromised organ function. Urgent intervention stands as imperative for TTP management [3]. This affliction can stem from an innate deficiency of the ADAMTS13 enzyme or an acquired deficit prompted by factors such as infections, malignancies, drugs, or immune anomalies [4]. While a classic TTP presentation entails a pentad of symptoms, comprising fever, thrombocytopenia, neurological manifestations, microangiopathic hemolytic anemia, and renal impairment, it is essential to recognize that most instances do not encompass all five symptoms [5]. Notably, dengue infection has been identified as a potential precipitant of TTP through its induction of an acquired ADAMTS13 deficiency. While the precise incidence remains elusive, a number of case reports have documented instances of TTP arising during dengue fever [6].

The primary therapy for TTP centers around plasma exchange therapy (PEX), whereas glucocorticoids assume the foremost role in treating ITP [7]. Although rituximab finds utility in the management of both ITP and TTP, it does not stand as the initial treatment of choice [7]. Albeit the co-occurrence of TTP and ITP in a single patient is an infrequent phenomenon, documented occurrences exist within select groups, including pregnancies, acquired immune deficiency syndrome (AIDS), and Sjogren's syndrome [8,9]. This case report chronicles the journey of a 53-year-old female patient previously diagnosed with ITP, who subsequently developed concurrent TTP precipitated by dengue infection.

## Case presentation

A 53-year-old female patient with a 10-year history of hypertension and immune thrombocytopenia (ITP) presented with a fever persisting for one week, accompanied by documented temperatures reaching up to 102°F. The patient encountered three episodes of gum bleeding and sought medical attention at a local hospital, where she received a diagnosis of severe anemia and thrombocytopenia. Subsequently, she was admitted, and a routine workup was conducted.

**Figure 1:**
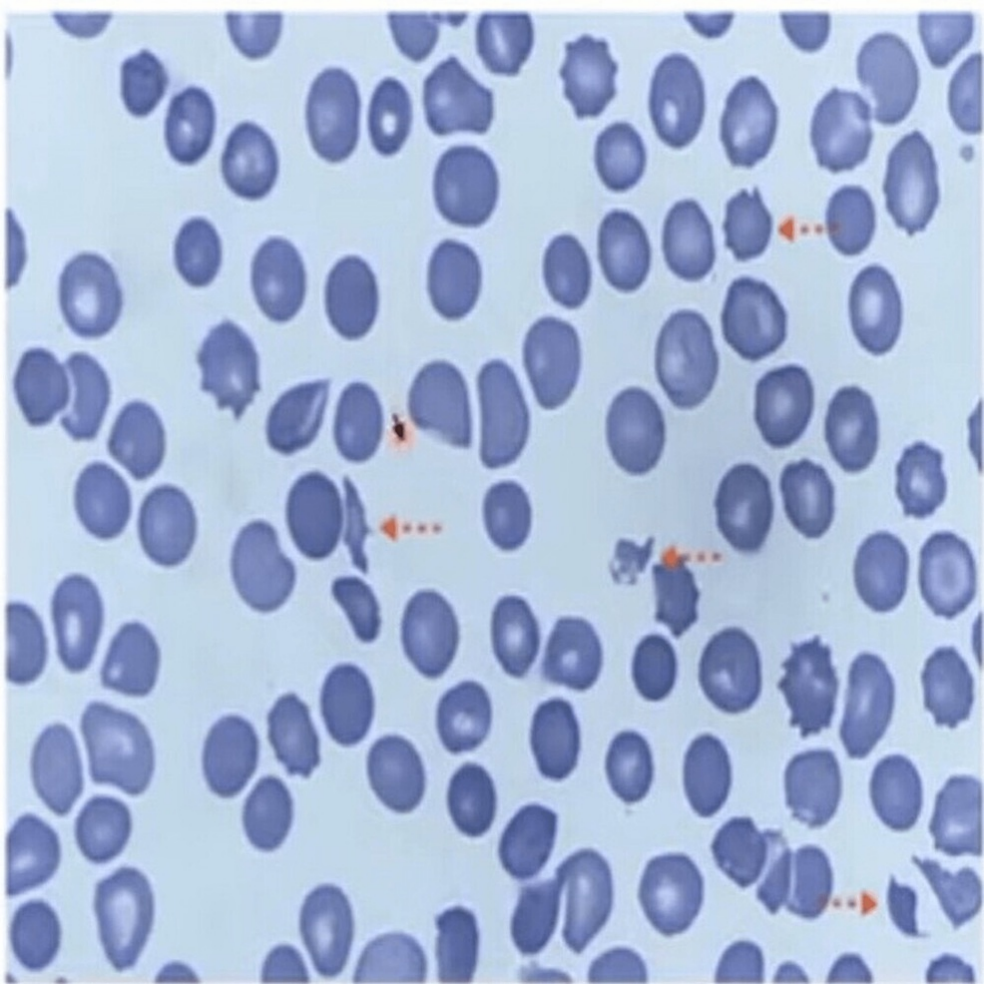
Peripheral blood smear (PBF) findings Peripheral blood smear showing irregularly shaped, jagged, fragmented RBC (schistocytes) suggestive of ongoing hemolysis.

Upon physical examination, pallor and an ecchymosis patch on the left shin area were observed. The patient had clear lung sounds, normal jugular venous pressure (JVP), and no hepatosplenomegaly. Initial laboratory findings indicated a hemoglobin (Hb) level of 7.7 g/dL, a platelet count of 18,000/cubic mm, and a total leukocyte count (TLC) of 5,700 cells/cubic mm. The dengue NS1 antigen tested positive at an outside hospital, and a dengue IgM test by enzyme-linked immunosorbent assay (ELISA) was performed at our hospital, yielding a positive result. In response to bleeding diathesis, the patient was administered a total of 12 units of random donor platelets (RDP) and two units of packed red blood cells (PRBC). Despite these interventions, the patient's Hb level continued to persistently decline from 7.7 g/dL at presentation to 4.4 g/dL, and the reticulocyte count increased to 15%. Peripheral blood film examination revealed the presence of schistocytes, as depicted in Figure 1. The lactate dehydrogenase (LDH) level was elevated at 2068 IU/L, while plasma and urine hemoglobin tests yielded negative results. Serological tests for HIV, hepatitis B, and hepatitis C were negative, and anti-nuclear antibodies (ANA) detected through indirect immunofluorescence assay (IFA) were also negative. G6PD and D-dimer levels, as well as serum electrolytes, renal function tests (RFTs), and liver function tests (LFTs), all fell within normal ranges.

Initially, the patient received an intravenous pulse dose of methylprednisolone (1 g) over a span of three days, as a response to the suspicion of a chronic ITP flare-up. Unfortunately, this intervention did not yield any improvement. Given the persistent hemolysis and thrombocytopenia, coupled with the ineffectiveness of intravenous steroids, a trial of intravenous immunoglobulin (IVIG) was initiated at a dosage of 2 mg/kg over the course of two days. Regrettably, this approach also proved to be ineffective. Subsequently, the patient experienced an episode of generalized tonic-clonic seizure, despite having no prior history of such seizures. No focal neurological deficits were identified during clinical assessment, and a fundus examination revealed grade 1 hypertensive retinopathy without the presence of papilledema. An MRI of the brain revealed no detectable abnormalities.

Given the emergence of this new neurological symptom and the suspicion of TTP, the PLASMIC score was calculated and found to be high (Table 1).

**Table 1:** PLASMIC score calculation for the patient The calculated PLASMIC score was 6, i.e., a high risk of severe ADAMTS13 deficiency.

Parameters	Finding	Score
Platelet count	18,000/cubic mm	1
Combined hemolysis variable	Reticulocyte counts at 15%, peripheral blood film examination revealed the presence of schistocytes, and lactate dehydrogenase (LDH) level was elevated at 2068 IU/L	1
Active cancer	Absent	1
Stem-cell or solid-organ transplant	Absent	1
Mean corpuscular volume	92 fL	0
International normalized ratio	1.0	1
Creatinine	0.9 mg/dL	1
PLASMIC score	6	
Risk group	High risk (more than 5)	

In consideration of the concurrent dengue infection and previous research indicating instances of dengue-mimicking relapse or progression of underlying hematological disorders, ADAMTS13 enzyme levels were subsequently measured [10]. These levels indicated 8% of normal activity, thereby confirming the diagnosis of TTP through the revelation of decreased ADAMTS13 enzyme activity. Following the confirmation of the TTP diagnosis, the patient underwent two cycles of plasmapheresis, leading to an improvement in her overall condition as well as positive changes in laboratory parameters such as hemoglobin, platelet count, and ADAMTS13 enzyme levels. Upon resolution of the hemolysis and thrombocytopenia, the patient was discharged and prescribed immunosuppressants along with oral steroids. A subsequent blood count assessment after one month showed a hemoglobin level of 10.9 g/dL and a platelet count of 200,000/mm3, with no recurrence of symptoms.

## Discussion

The management of ITP typically involves initiating treatment with high-dose steroids, followed by a tapering dose. Additional treatment options include rituximab, IVIG, or thrombopoietin receptor agonists. In some cases, splenectomy or the use of immunosuppressive agents or antibodies targeting CD40-CD154 may be considered [11]. Our patient also initially responded well to steroid treatment upon the first diagnosis of ITP.

However, when the patient did not exhibit improvement with steroid and IVIG therapy and developed new central nervous system (CNS) symptoms, the possibility of concurrent TTP triggered by dengue infection was considered. There have been several reported cases of TTP occurring after dengue infection. Despite the absence of the characteristic pentad of TTP, the persistence of thrombocytopenia, hemolysis, and a positive dengue IgM result, along with a high PLASMIC score [9], led us to suspect the coexistence of TTP and ITP. To confirm the diagnosis of TTP, ADAMTS13 enzyme levels were measured and found to be decreased. Various conditions, including infections, connective tissue diseases, pregnancy, and malignant tumors, can lead to acquired TTP [12,13]. However, our patient did not exhibit typical features of connective tissue diseases, and significant renal impairment was ruled out based on negative ANA/C3/C4 test results.

TTP is a medical emergency that demands immediate attention due to its potentially life-threatening nature. Therapeutic plasma exchange (PEX) is the recommended first-line therapy for TTP, as it replenishes deficient ADAMTS13 enzyme levels [7]. Steroids can be used as immunomodulators alongside PEX, targeting ADAMTS13 enzyme autoantibodies [14]. Plasmapheresis effectively corrected our patient's hemolysis and thrombocytopenia. PEX functions by removing ADAMTS13 enzyme inhibitors, unusually large von Willebrand factor multimers (UL-VWFM), and inflammatory cytokines responsible for the release of UL-VWFM from vascular endothelial cells. Additionally, PEX replenishes ADAMTS13 enzyme and normal-sized vWF multimers, which are crucial for normal hemostasis.

Glucocorticoids are commonly included as an adjunct therapy in the management of TTP, expediting recovery by reducing the production of ADAMTS13 enzyme inhibitors, decreasing cytokine production, and mitigating autoantibody-mediated clearance of ADAMTS13 enzyme. According to the 2020 guidelines of the International Society on Thrombosis and Haemostasis (ISTH), glucocorticoids are recommended in combination with PEX as the initial treatment for TTP [15]. However, it should be noted that glucocorticoid monotherapy is ineffective in managing TTP, as demonstrated by a study where approximately half of the patients who received glucocorticoid monotherapy did not show improvement [16]. Another study comparing prednisone to cyclosporine in terms of increasing ADAMTS13 enzyme activity and suppressing anti-ADAMTS13 antibodies found that prednisone was superior to cyclosporine [7]. While glucocorticoid monotherapy is not effective in TTP, it is the first-line treatment for ITP, with ITP patients generally responding favorably to glucocorticoid therapy, as our patient did initially upon being diagnosed with ITP. The addition of rituximab to corticosteroid and PEX therapy has been shown to improve outcomes in refractory TTP [17].

Caplacizumab, an anti-vWF immunoglobulin targeting the A1 domain of vWF, prevents the interaction of vWF with the platelet glycoprotein Ib-IX-V receptor [18]. The use of caplacizumab in conjunction with standard treatment for acquired TTP has demonstrated promising results, including early recovery of platelet count to normal levels and decreased recurrence rates [19]. The recombinant ADAMTS13 enzyme is an emerging treatment for congenital TTP and has also been explored for acquired TTP [20].

In our patient's case, she responded well to plasmapheresis, undergoing two cycles, which resulted in improvements in hemoglobin and platelet count, as well as clinical recovery.

## Conclusions

This case report underscores the significance of considering TTP in patients presenting with thrombocytopenia and microangiopathic hemolytic anemia, particularly when standard ITP treatment proves ineffective, especially in the presence of known triggers like dengue infection, as observed in our case. Timely diagnosis and treatment involving PLEX and immunomodulators can substantially enhance patient outcomes for TTP. Clinicians need to be attentive to the diagnostic complexities associated with TTP, and the possibility of TTP should be contemplated in instances of unexplained thrombocytopenia and microangiopathic hemolytic anemia.

It is worth noting that the classic pentad of TTP may not manifest in all patients, thus maintaining a heightened level of suspicion is crucial for those with thrombotic microangiopathy. The use of the PLASMIC score can serve as an adjunct to strengthen the diagnostic consideration of TTP. Moreover, given the heightened association between dengue infection and increased TTP incidence, physicians should remain vigilant about this potential complication. Since TTP is an acute, life-threatening condition, early treatment is imperative, necessitating a high index of suspicion even in rare scenarios.
